# Cases of Guinea Worm Disease Among Al Sabah Children Hospital Attendees from January to December 2022, South Sudan

**DOI:** 10.24248/eahrj.v8i3.803

**Published:** 2025-01-30

**Authors:** Ezbon WApary, Albino G Atak, Jacob Y Awuol, John M Adut, Gai Y Pech, Jackson M Ayii, Philip M Mayar, Akway M Cham

**Affiliations:** a School of Medicine, University of Juba, South Sudan.

## Abstract

**Background:**

Guinea worm disease carries health risks with potential effects on social and economic wellbeing of an individual and community. Emergence, the fact of manually removing the worm, is slow, painful, and disabling and therefore, it has a serious adverse socioeconomic outcome on the health, agricultural productivity, and school attendance of affected population. This study was conducted to identify case of Guinea worm among children attending Al Sabah Children Hospital, South Sudan.

**Methodology:**

The study extracted register data which covered the period from January to December 2022. Using a systematic random sampling, 422 children were selected from the hospital’s Statistics Department of Al Sabah Hospital. Descriptive data analysis was performed using SPSS Statistics software.

**Results:**

Of the children who received hospital services, the vast majority (76.3%) were children <5 years old, and more than half were females. None of the sampled children had Guinea worm infection.

**Conclusion:**

In 2022, none of the attendees of the Al Sabah Children Hospital was diagnosed with Guinea worm infection. But this does not mean that the country is free of the disease as the study design did not allow to establish true prevalence.

## BACKGROUND

Dracunculiasis, also called Guinea worm disease (GWD), is a neglected tropical disease (NTD) caused by a nematode (Dracunculus medinensis). The name originally came from its prevalence in the Gulf of Guinea. For the last decades, dracunculiasis was named as “the disease of the empty granary” due to the difficulties individuals faced in going to work in fields or to school when affected by this disease.^1–4^

Humans contract Guinea worm disease (GWD) by drinking unfiltered water from wells and other stagnant water sources infested with, “water fleas” too small to be seen with the naked eye. Guinea worm larvae are ingested by copepods which are ingested, in turn, by people who drink the contaminated water. In the human host, the copepod is dissolved by gastric juice, releasing Guinea worm infective larvae. Once in the stomach, the larvae penetrate the gastric or intestinal mucosa and, after a period in the abdominal cavity, migrate up to the connective tissue where Dracunculus larvae mature to adult worms and, after mating, the male worms die, and female worms mature and acquire a cylindrical, white and smooth body.^1,2,5^

The tip of the tail is pointed forming a blunt hook. Female worms take 9 to 14 months to reach adult form and can measure up to 1 m in length. Approximately one year after infection, female worms migrate through the subcutaneous tissue, reach the surface and emerge from the skin, often at the lower extremities. Before they emerge, they create a painful and itchy blister on the skin site. Affected individuals normally seek pain relief by dipping the lower limbs in cold water, an action that leads to the rupture of the blister and the emergence of the worm from the skin. In this phase, the first stage larvae are released into the water and are then ingested by the copepods, reaching the final stage and re-starting their life cycle.^1,2,5^

Worldwide, in the 1940s, an estimated 48 million people were affected by GWD in Africa, the Middle East, and India, while in the 1980s, only 3.5 million cases per year were reported in 20 countries worldwide, including 17 in Africa. The number of reported cases dropped below 10,000 for the first time in 2007, falling further to 542 cases in 2012, 54 cases in 2019, and 27 cases in 2020 (of these cases, 1 in Angola, 12 in Chad, 11 in Ethiopia, 1 in Mali, 1 in South Sudan, and 1 in Cameroon). The six other countries are either endemic (Angola, Chad, Ethiopia, South Sudan, and Mali) or are in the pre-certification phase (Sudan). 15 Guinea worm cases were reported in 2021.^1,6,9^ In 2022, 4 countries reported a total of 13 human cases of dracunculiasis, namely, Chad (6 cases), Ethiopia (1 case), South Sudan (5 cases) and the Central African Republic. These 5 human cases of dracunculiasis were from 3 villages in 3 counties (Awerial, Juba and Tonj East), of which 3 were contained.^7^ Yearly, the GWD cases have been continuously reported since the 1990s, but the global burden of disability due to GWD has not previously been amounted in the global burden of diseases, injuries, and risk factors study in 1990, a total of 624,000 cases were reported globally; in 2016, only 25 cases were reported across four remaining endemic countries of (Chad, Ethiopia, Mali, and South Sudan). If the global campaign is successful, Guinea worm could be the second human disease in history eradicated by direct public health interventions after Wuchereria bancrofti fiariasis,^5,8^ 199 countries areas, and territories have been certified as free from dracunculiasis.^1,4,6,9^

With only five countries currently affected by dracunculiasis (Angola, Chad, Ethiopia, Mali, and South Sudan), achievement of eradication is within reach. This is due to civil unrest, insecurity, and lingering epidemiologic and zoologic questions.^9^ The four nations with the remaining Guinea worm caseload, that is, South Sudan, Ethiopia, Chad, and Mali have endorsed target to interrupt the transmission of the disease.^10^ The number of reported cases reduced from 900 000 from 20 countries in 1989 to 542 from four countries at the end of 2012. All but 21 of 542 cases of Guinea worm reported globally in 2012 (96.1%) were from South Sudan. Of South Sudan’s 79 counties, 71 were Guinea worm-free by 2012, compared with 57 Guinea worm-free counties in 2009. In 2012, 81% (410) of all Guinea worm cases in South Sudan were concentrated in only one county – Kapoeta East, in Eastern Equatoria State. Of these cases, 66% were contained, including 236 newly diagnosed patients who were voluntarily confined to a case containment center.^10^

After reporting no cases of dracunculiasis for the first time in 2017, South Sudan reported 10 human cases in 2018. Eight patients were young cattle herders from migratory communities in recently pacified areas that had experienced chronic communal violence and population displacements in recent years. Extreme mobility of cattle herders and others is a special challenge in addition to sporadic insecurity. South Sudan reported no cases in January–June 2019, compared with four cases in January–June 2018. By December 2018, South Sudan’s GWEP had 2,165 villages under active surveillance. In January 2018, the reward for reporting a case of dracunculiasis increased to US$400 from US$140. Of 1,694 residents in villages under active surveillance, 72% of the respondents knew of the reward for reporting an infected person.^8,9^

Diagnosis of GWD is mainly clinical and it consists of observation of the worm emerging from the blister. The blisters cannot be distinguished from other common skin lesions, such as bacterial infections or diabetic foot-related conditions. The diagnosis can be formulated only when the female worm emerges, typically wrapping around a stick. During worm spillage, the diameter of the nematode should be assessed, since bodies that are smaller than 2 mm represent a risk factor for worm body rupture. Active larvae can be obtained by immersing the protruding adult female in a small tube or container with water. First-stage larvae, with their characteristic pointed tails, can then be observed under a microscope.^1,11^

Despite the simplicity of diagnosis, GWD can be misdiagnosed due to the non-specificity of initial symptoms. In addition, in countries where the two parasites are co-endemic, differential diagnosis with Onchocerca spp is needed. Radiological diagnosis is also possible, even if it generally represents an occasional finding. Case reports of breast location are described during mammogram screening. A typical radiological finding of GWD is the calcification sign. Calcification occurs once the gravid female dies inside the soft tissue.^1,11^

There is no vaccine to prevent dracunculiasis, and eradication relies on case containment and prevention of water contamination and other interventions to prevent infection, health education, water filtration, treatment of unsafe water with an organophosphate larvicide, and provision of safe drinking water.^4,7^ There is no specific chemotherapy for GWD. Diagnosis occurs at emergence and treatment is limited to case management to avoid secondary bacterial infections by using sterile bandages, topical antibiotic ointment, and treatment with anti-inflammatories.^5^

First-line treatment of Guinea worm infection consists of removing the female worm when it comes out of the skin and pulling it out gently. This is to avoid rupture or returning itself inside the wound. Usually, a gauze or a small stick is used to allow the worm to roll around it, continuing to exert some traction to bring it out. Two actions facilitate the exit of the worm: dipping the affected body part in a bucket with water and squeezing the bump to empty the adult worm from the larvae, so that it is thinner and can exit from the wound more easily.^1^

Support therapies such as anti-inflammatory drugs and painkillers can be used to reduce oedema and pain. Along with frequent dressings with antiseptic solutions, antibiotic ointments may be applied to blisters to avoid wound superinfections. Until the whole worm body has been pulled out, the wound must be covered with medicated gauze and, until successful eradication, an infected person is not allowed to enter drinking water sources. Tetanus vaccination is recommended. GWD-specific vaccines are not available.^1^

Guinea worm disease carries health risks with potential effects on social and economic wellbeing of an individual and community. Emergence, the fact of manually removing the worm, is slow, painful, and disabling and therefore, it has a serious adverse socioeconomic outcome on the health, agricultural productivity, and school attendance of affected population.^1^

Despite the fact that Guinea worm disease is identified as a serious health impacts with economic and academic problems all over the world, the number of cases of Guinea worm among children attending Al Sabah Children hospital is not known in South Sudan. Therefore, the aim of this study was to identify cases of Guinea worm disease among children attending Al Sabah hospital in South Sudan. Findings from this study contribute to the information source to be used by the various organizations and government for the purpose of advancing the ways of preventing Guinea worm.

## METHODS

### Study Area

The study was conducted at Al Sabah Children Hospital. This hospital was established by the Kuwait government in 1983 and is a government hospital under the Ministry of Health, Central Equatoria state. It is located along Unity Avenue, Juba. Early in 2017, the department of paediatrics and child Health at Juba Teaching Hospital was moved to this hospital. The purpose was to provide optimal care to infants, children and adolescents in a specific environment where parents are admitted and where the special needs of children are catered. Currently, the hospital is the only functional paediatric hospital in South Sudan, receiving patients from all parts of the country and giving clinical training to students from both public universities and private institutions.

### Study Design and Methods

This study employed quantitative cross-sectional study design to determine the prevalence of Guinea worm disease among children attending Al Sabah Children Hospital of South Sudan. It extracted the data of the children from the register of January to December 2022 from Statistics Department of the hospital.

### Sample Size Calculation

This sample was obtained by using Cochran’s formula: where n is the sample size, is the abscissa of the normal curve that cuts off an area at the tails (the desired confidence level of 95%), is the desired level of precision, is the prevalence and is .10% was added for marginal errors. ^12^ Thus, the sample size was 422 records.

### Sampling Procedure

This study used systematic random sampling technique. This included getting a sample by selecting each 6^th^ from the list of January to December 2022 registers. If the record was incomplete, the research team would select next number. This sampling method was performed until the final sample size reached.

### Inclusion and Exclusion Criteria

All records of children who were between 1–17 years and attended the hospital during the study period were eligible for this study. And those who were >1 or <18 years were excluded.

### Study Variables

The variables included total number of children attended at the hospital during the study period, number of sampled children, number of children diagnosed with Guinea Worm. Other variables were demographic characteristics, such as age of child, sex, place of residence and among others.

### Data Processing and Analysis

Using EpiData Manager version 4.6 (CDC, USA), the software questionnaire was designed, prepared and checked (legal range, jump, must enter value and labels). This designing process was performed after the data had been cleaned up for omission and errors during the data collection process. Afterwards, the data were entered into the EpiData so as to form database (rec) that was exported into statistical package for social science (SPSS statistics version 26, IBM, USA) data base (spv) for analysis phase. This statistical software was used in order to check for descriptive statistics, for example, frequencies and percentages; and inferential statistics, such as Chi-square.

### Ethical Consideration

The study has received approved letter with reference no. 9/2023 from the School of Medicine, University of Juba to conduct the cross-sectional survey on the Prevalence of Guinea Worm Disease among Children Attending Al Sabah Children Hospital of South Sudan. This letter was put forward to the Ministry of Health, Central Equatoria State and the Medical Director of the hospital to which gave permission for data to be collected.

## RESULTS

Table 1 illustrates statistical descriptive analysis of demographic characteristics of children attending Al Sabah Hospital during the study period. A total of 422 records of the children were extracted from registers of Al Sabah Hospital. Of the 422 children who were attending at the hospital services, 76.3% were children <5 years old, and the more than half were female. These children came mainly from blocks of Juba City Council residences. While Kator block accounted for 31.0%, followed by Munuki block and then Juba block.

**Table 1:** Demographic Characteristics of Children Attended Al Sabah Hospital During Study Period

Variable	n=422	%
Age		
< 5 years	322	76.3
≥ 5 years	100	23.7
Sex		
Male	205	48.6
Female	217	51.4
JCC Blocks		
Kator	131	31.0
Munuki	100	23.7
Juba	74	17.5
Others	117	27.7

JCC: Juba City Council

None of the children who attended Al Sabah Hospital during study period was diagnosed with Guinea worm infection. Malaria (73.2%) was the most frequently diagnosed infection, followed by sepsis (Table 2).

**Table 2:** Proportion of Children Diagnosed with Guinea Worm and Other Infections During Study Period

Variable	n=422	%
Diagnoses		
Guinea worm	0	0
Malaria	311	73.7
Sepsis	49	11.6
URTI	21	5.0
Anaemia	8	1.9
Sepsis	8	1.9
Pneumonia	6	1.4
Septicaemia	6	1.4
Typhoid	5	1.2
Diarrhoea	2	0.5
Gastroenteritis	2	0.5
Dermatitis	1	0.2
Gastritis	1	0.2
Roundworm	1	0.2
UTI	1	0.2

## DISCUSSION

### Proportion of Al Sabah Children Hospital Attendees Diagnosed with Guinea Worm

None of the children attending Al Sabah Hospital was diagnosed with Guinea worm. In other words, there was no case of Guinea worn reported between January and December 2022 at Al Sabah Children Hospital. In 2012, a total of 27 human cases were reported. Of those, 1 case was in South Sudan, 1 in Angola, 12 in Chad, 11 in Ethiopia, 1 in Mali, and 1 in Cameroon.^1,13^ In 2022, a total of 13 human cases of dracunculiasis were reported from 4 countries (Chad 6 cases, Ethiopia, 1 case, South Sudan 5 cases, and I case in Central African Republic). In South Sudan, the 5 human cases of dracunculiasis were from 3 villages in 3 counties (Awerial, Juba and Tonj East).^7^ Whereas the 3 cases were contained,^7^ the outcome of other two cases were unclear. While Al Sabah Children Hospital is the only paediatric hospital in South Sudan, those cases might have never been referred to this hospital. Furthermore, South Sudan reported 10 human cases in 2018. South Sudan reported no cases in January to June 2019.^8,9^

While the risk factors of Guinea worm disease includes sex, residential areas, level of education, occupation, source of drinking water and among others, the statistically association of these factors were not performed. This was because the prevalence of Guinea worm was 0%. The disease occurs most common among young adults 15 to 45 years old. Regarding to the profession, the farmers, herders, those fetching drinking water for households may be more likely to become infected.^1^ The prevalence of the disease depends on rain patterns and climate that is in arid areas, the disease tends to spread intensely in the rainy season, when there is an increase availability of surface water while in wet areas, the disease prevalence tends to be more, when there are limited drinking sources, such as stagnant water collection points like wells and cisterns.^1^

Guinea worm is one of the most common filariases that has been widespread in countries with low economic status in tropical regions of Africa and South Asia, where it has been associated with low levels of education.^1^ The disease is common in rural communities of countries with low income, which depend on open surface water for domestic use. It is one of most neglected causes of disability in rural areas of Africa, south-west Asia, and India, whose survival depends on such water. GWD can affect people of all ages but is more common in young adults aged 15 to 45, with no difference in prevalence between males and females cases.^1^

### Study Limitations

This study focused only on registers of children attending Al Sabah Children Hospital. Investigation on Guinea worm infection is not routinely performed, and therefore some cases might have been missed. True prevalence cannot be established using the type of data source used in this study.

## CONCLUSION

In 2022, none of the attendees of the Al Sabah Children Hospital was diagnosed with Guinea worm infection. But this does not mean that country is free of the disease as the study design does not allow to establish true prevalence.
